# Interaction between cognitive reserve and age moderates effect of lesion load on stroke outcome

**DOI:** 10.1038/s41598-021-83927-1

**Published:** 2021-02-24

**Authors:** Roza M. Umarova, Lena V. Schumacher, Charlotte S. M. Schmidt, Markus Martin, Karl Egger, Horst Urbach, Jürgen Hennig, Stefan Klöppel, Christoph P. Kaller

**Affiliations:** 1 Faculty of Medicine, University of Freiburg, Freiburg im Breisgau, Germany; 2 Department of Psychiatry and Psychotherapy, Medical Center, University of Freiburg, Freiburg im Breisgau, Germany; 3 BrainLinks-BrainTools Cluster of Excellence, University of Freiburg, Freiburg im Breisgau, Germany; 4 Department of Neurology, University Hospital Bern and University of Bern, Bern, Switzerland; 5 Medical Psychology and Medical Sociology, Faculty of Medicine, University of Freiburg, Freiburg im Breisgau, Germany; 6 Center for Geriatric Medicine and Gerontology, Medical Center, University of Freiburg, Freiburg im Breisgau, Germany; 7 Department of Neuroradiology, Medical Center, University of Freiburg, Freiburg im Breisgau, Germany; 8 Medical Physics, Department of Radiology, Medical Center, University of Freiburg, Freiburg im Breisgau, Germany; 9 University Hospital of Old Age Psychiatry Bern, Bern, Switzerland

**Keywords:** Stroke, Outcomes research, Cognitive neuroscience, Neural ageing, Neuro-vascular interactions

## Abstract

The concepts of brain reserve and cognitive reserve were recently suggested as valuable predictors of stroke outcome. To test this hypothesis, we used age, years of education and lesion size as clinically feasible coarse proxies of brain reserve, cognitive reserve, and the extent of stroke pathology correspondingly. Linear and logistic regression models were used to predict cognitive outcome (Montreal Cognitive Assessment) and stroke-induced impairment and disability (NIH Stroke Scale; modified Rankin Score) in a sample of 104 chronic stroke patients carefully controlled for potential confounds. Results revealed 46% of explained variance for cognitive outcome (p < 0.001) and yielded a significant three-way interaction: Larger lesions did not lead to cognitive impairment in younger patients with higher education, but did so in younger patients with lower education. Conversely, even small lesions led to poor cognitive outcome in older patients with lower education, but didn’t in older patients with higher education. We observed comparable three-way interactions for clinical scores of stroke-induced impairment and disability both in the acute and chronic stroke phase. In line with the hypothesis, years of education conjointly with age moderated effects of lesion on stroke outcome. This non-additive effect of cognitive reserve suggests its post-stroke protective impact on stroke outcome.

## Introduction

Prediction of stroke outcome remains challenging due to large inter-individual variability, which is expected to increase further as ageing of the stroke population continues and concomitant neurodegenerative changes and multi-morbidity are rising^1^. Plenty of factors have been reported to impact stroke outcome: Demographic factors (e.g. older age, female sex, lower educational attainment) and clinical and stroke characteristics (initial stroke severity, lesion load) constitute significant predictors for poorer post-stroke cognitive functioning^2–4^. However, as of yet, all these factors were mainly investigated in isolation, neither controlling for potential confoundings nor assessing mutual interactions. For example, women tend to be affected by stroke at an older age than men^5^. At the same time, women had for a long time in general lower educational attainment than men due to the socio-demographic developments in the past. Thus, comprehensively controlling for such interdependencies is mandatory for achieving unbiased insights into the factors’ genuine contribution to post-stroke cognitive outcome.

**Figure 1:**
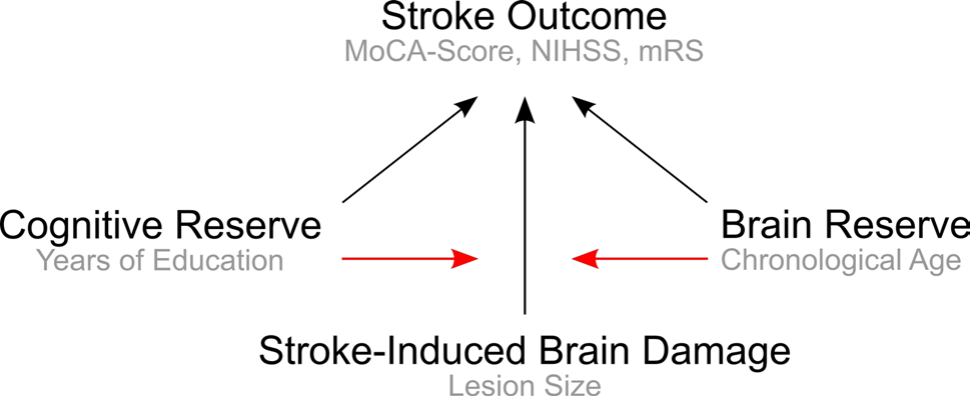
Impact of cognitive and brain reserve on effects of stroke-induced brain damage on stroke outcome. Black arrows indicate already established simple effects of key determinants of stroke outcome, whereas red arrows indicate their hypothesized mutual interaction. The respective proxies used in the present analyses are annotated in grey font. Note that for sake of clarity the illustration focuses only on the three-way interaction. The figure was created using Inkscape software, version 1.0 https://inkscape.org/.

In the context of neurodegenerative diseases, inter-individual variability in susceptibility to pathology is explained by concepts of brain reserve and cognitive reserve: individuals with higher reserve can tolerate more pathology and maintain function^6^. Brain reserve is originally defined by quantitative neural characteristics (e.g. total intracranial volume)^6,7^. Cognitive reserve is defined as cognitive capacities acquired via lifetime intellectual activities, occupational-educational history, and other environmental factors, which shape the brain’s network efficiency, processing capacity, and flexibility^6^. Cognitive reserve is a dynamic feature of brain function proposed to moderate the impact of pathology on performance^6^. Moreover, cognitive reserve is suggested to shape the brains' ability to compensate for injury by facilitating neural compensation^6^. Though cognitive reserve is developed via engagement in complex lifestyle activities, these activities do not accurately represent the cognitive reserve construct—they are merely proxy measures of it. We recently suggested that the concepts of brain and cognitive reserve might constitute a theoretical framework to capture inter-individual variability and to improve prediction of outcome in stroke: Stroke outcome might accordingly result from the *interaction* between pre-stroke brain reserve and cognitive reserve and severity of stroke damage (Fig. 1)^8,9^. In the present observational-cohort study, we addressed this hypothesis using proxies easily available in the routine clinical practice. We used age as a simple and global coarse proxy of brain reserve as it sufficiently reflects individual neuroanatomical differences correlating with volumetric brain characteristics^10^, cortical atrophy and leukoaraiosis^11,12^, and with age-associated changes in neuroplasticity^13^. Years of education were used as a proxy of cognitive reserve^7^. Of note, years of education are not a direct measure of cognitive reserve, as well as other proxies used to operationalize this construct. We used lesion size as a global measure of the severity of stroke-induced brain damage^14–16^. We applied the model to predict (i) post-stroke cognitive outcome, and (ii) post-stroke impairment and disability. For disentangling the individual contributions of these three determinants of stroke outcome, we (i) applied a paired-matching approach^17,18^ to remove the confounds between them and (ii) statistically accounted for their interplay by explicitly modeling the respective two- and three-way interactions^19^.

## Methods

### Subjects

Chronic stroke patients were recruited between October 2013 and October 2016 from the Department of Neurology at the University Medical Center Freiburg and tested in the chronic phase of stroke (> 5 months post-stroke). Main inclusion criteria for the present analyses constituted a first-ever ischemic stroke in the middle cerebral artery territory. Exclusion criteria in the acute phase were (i) an age over 90 years; (ii) conditions compromising study participation and examination (e.g. low consciousness or arousal level, general MRI contraindications); (iii) pre-stroke neurological or psychiatric conditions compromising data interpretation (pre-stroke cognitive impairment, previous stroke, structural brain lesions besides stroke etc.); and (iv) illiteracy^20^. Every eligible patient was asked to participate and, once consented, tested at the Department of Neurology. A total of 153 (mean age ± SD, 64.4 ± 13.8 years; 52 female) patients conformed to these criteria and underwent examination in the chronic stroke phase. All patients were treated according to the Guidelines of the German Neurological Society. This study was compliant with the declaration of Helsinki; full written informed consent was obtained from all patients or their legal guardian accordingly. The Ethics Committee of the University Medical Centre Freiburg approved the study.

### Neurocognitive and clinical assessments

Assessment of post-stroke cognitive outcome in the chronic stroke phase (on average 17.0 ± 18.5 months post-stroke, range 5.0–72.5 months) comprised the Montreal Cognitive Assessment (MoCA)^21^ as the main variable of interest, which was adapted for the German-speaking population (http://www.mocatest.org/pdf_files/test/MOCA-Test-German2.pdf) and is applicable in stroke patients^22,23^. In addition, the NIH Stroke Scale (NIHSS) and the modified Rankin Scale (mRS) were available in the acute stroke phase (from the routine clinical examination at discharge on average 9.5 ± 4.1 days post-stroke, range 4–21 days) and at the chronic stroke phase (on average 17.3 ± 18.5 months post-stroke, range 5.0–72.5 months). Table 1 provides a descriptive overview of the dependent variables in the overall sample as well as in the matched sample (see also below).

**Table 1:** Descriptive overview of stroke outcome measures in the overall and matched samples.

Variable	Assessment	Overall Sample (n = 153)			Matched Sample (n = 104)		
		n	Mean ± SD	Range	n	Mean ± SD	Range
MoCA	Chronic	153	23.1 ± 5.1	7–30	104	23.8 ± 5.0	8–30
mRS	Acute	145	1.8 ± 1.4	0–5	97	1.7 ± 1.4	0–5
	Chronic	145	1.3 ± 1.2	0–5	100	1.1 ± 1.0	0–5
NIHSS	Acute	152	2.7 ± 3.1	0–16	103	2.5 ± 2.9	0–16
	Chronic	151	1.3 ± 1.9	0–11	103	1.2 ± 1.8	0–11

*MoCA* Montreal Cognitive Assessment, *mRS* modified Ranking Scale, *NIHSS* National Institutes of Health Stroke Scale, *SD* standard deviation.

### MR imaging and lesion mapping

In the acute stroke phase, all participants were administered MRI-examination according to the protocol and post-processing as previously described^20^. Lesion mapping analysis was performed semi-automatically as previously described based on the MRI imaging acquired 2–3 days post-stroke^20,24^. At this time point, the ischemic damage is completely apparent in the diffusion-weighted and T2-/fluid attenuation inversion recovery (FLAIR) images. In the chronic phase, MRI including FLAIR sequences was acquired to exclude new infarcts.

### Matching procedure

Data exploration revealed that fewer years of education were associated with older age and female sex reflecting the last century’s historical and social developments in Germany and Western Europe. To balance this confound, which prevented the analysis of the proxy’s independent impact, we performed a multi-dimensional matching approach with a two-step procedure. First, we divided the sample along its median of 12 years of education into two subsamples comprising patients with a higher (> 12 years of education; n = 68) and a lower (≤ 12 years of education; n = 85) educational attainment. Afterwards, an algorithm selected pairs of patients from these two subgroups with the same sex and the least differences in age and lesion size in terms of the Mahalanobis distance^25^ in the multi-dimensional space spanned by the three variables. As a cutoff for the optimal match, we considered a maximum Mahalanobis distance around 1 and an increasing steepness in the distance between adjacent pairs of patients, which together resulted in the final selection of n = 52 pairs with least Mahalanobis distances^17^.

### Statistical analyses

Regression analyses with interaction effects were conducted using the Statistics toolbox for Matlab (R2018a; The Mathworks, Inc., Natick, MA, USA) entering either the MoCA scores (linear regression) or the dichotomized clinical outcome scores (logistic regression on NIHSS and mRS) as criterion variable. Lesion size, years of education and chronological age were entered as predictor variables and mean centered before computation of product terms, and their potential two-way and three-way interactions were explicitly modeled and analyzed. We controlled for the effect of total brain volume as another important proxy of brain reserve, but we did not introduce it additionally in the statistical model to avoid the problem of oversampling. Moreover, the impact of total brain volume and brain atrophy on post-stroke cognitive functioning and stroke outcome is established (IST-3 collaborative group, 2015; Sagnier and Sibon, 2019; Pedraza et al*.*, 2020; Schirmer et al*.*, 2020).

We applied a logistic regression analysis on dichotomized clinical outcome scores given rank ties and the non-gaussian distribution of these clinical scores. Dichotomization of each individual clinical score into two preferably equally sized classes separated patients into those with better (class 1) and poorer (class 2) outcome (acute NIHSS, class 1: NIHSS-score ≤ 1, class 2: NIHSS-score > 1; chronic NIHSS, class 1: score = 0, class 2: score ≥ 1; acute mRS, class 1: mRS ≤ 1, class 2: mRS > 1; chronic mRS, class 1: mRS ≤ 1, class 2: mRS > 1). For the chronic mRS, class 1 was however larger (n = 73) than class 2 (n = 23) due to low variance. Lesion overlays and analyses of lesion distributions were conducted using MriCroN^26^.

## Results

A sample of 153 patients with first-time ischemic stroke in the territory of the middle cerebral artery was included in the study. Each patient’s individual cognitive and brain reserve were approximated by years of education and age, respectively. Explorative analysis revealed that both age and years of education correlated with chronic stroke outcome as measured by the MoCA (r = − 0.428, p < 0.001; r = 0.379, p < 0.001 correspondingly) and with the routine clinical scores, i.e. chronic NIHSS (τ = 0.104, p = 0.046; τ = − 0.202, p = 0.001) and chronic mRS (τ = 0.169, p = 0.004; τ = − 0.200, p = 0.001). However, fewer years of education were considerably confounded in the present sample with older age (r = − 0.313, p < 0.001) and female sex (r = − 0.296, p < 0.001) that prevented the analysis of the independent impact of both variables.

### Matching and sample characteristics

**Figure 2:**
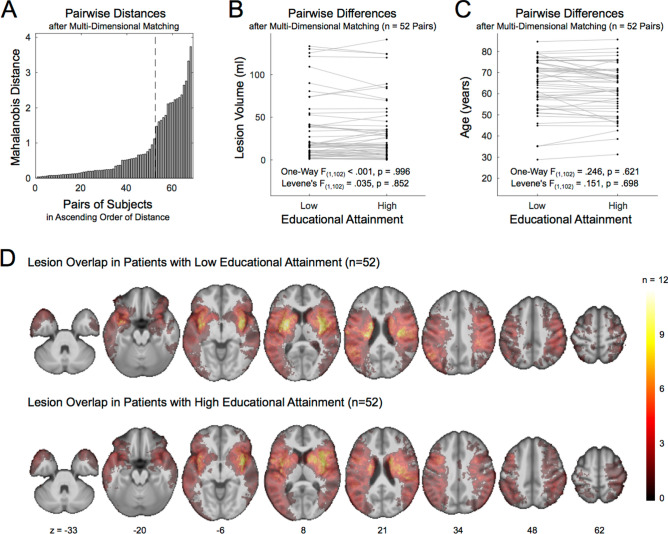
Matching of pairs of patients with high vs. low educational attainment used to control for potential confoundings. (**A**) Mahalanobis distances of pairs of patients with high vs. low educational attainment after multi-dimensional matching for lesion size, age, and sex. The broken line indicates the bend in distances after selected 52 pairs. The individual 52 pairs of patients with high vs. low educational attainment were comparable in lesion size (**B**) and age (**C**) as the multi-dimensional matching approach yielded both a minimized between-group difference (one-way ANOVA) and a maximized variance homogeneity (Levene’s test) between groups. Individual pairs are connected by gray lines. (**D**) Lesion distribution and overlap for patients with high and low educational attainment: Both groups were comparable in their lesion load (Liebermeister test, pFDR < 0.05). Colors indicate the lesion overlap. MNI z-coordinates are given for the slices. The figure was created using Matlab software, version 2018a https://www.mathworks.com/products/matlab.html and MRIcron, version 12.12.2012 https://www.nitrc.org/projects/mricron.

To avoid any bias in the subsequent analyses and to unequivocally separate the predictive values of age and years of education on stroke outcome, we applied a multi-dimensional matching approach^17^ and identified pairs of patients with different level of education but same sex and the least differences in age and lesion size in terms of the Mahalanobis distance^25^ in the multi-dimensional space spanned by the three variables (Fig. 2A). The matching approach resulted in 52 pairs of patients (63.3 ± 12.1 years; 32 female) who were of an almost identical age (p = 0.621; Fig. 2B), same sex (p > 0.999), and had stroke lesions of an almost identical size (p = 0.996; Fig. 2C), but significantly differed in years of education (p < 0.001). An overview of the sample characteristics of the resulting groups of stroke patients with low and high cognitive reserve is provided in table e-1. Additional analyses ensured that groups did not significantly differ with respect to the lesion anatomy (Liebermeister test, pFDR > 0.05, Fig. 2D), the duration of acute hospitalization, time of testing, stroke lateralization and the application of thrombolysis (highest t = 1.973, lowest p = 0.236) as well as the total intracranial volume (p = 0.404) and total brain volume (p = 0.609). In the matched sample (n = 104), years of education did not correlate with the demographic and clinical characteristics listed in table e-1 (0.022 <|r|< 0.144, 0.824 > p > 0.145). After multi-dimensional matching, the matched sample was hence free of confounds that were present between the key variables of interest (i.e. lesion size, age, years of education) in the overall sample of 152 patients (see above). For sake of statistical power in investigating potential interaction effects, the subsequent analyses were based on years of education as a continuous measure and not a dichotomous grouping of patients with high vs. low educational attainment.

### Prediction of post-stroke cognitive outcome

Prediction of post-stroke cognitive outcome was assessed using a linear regression analysis with patients’ MoCA scores at the chronic stroke phase as the criterion variable (i.e. as measure of global cognitive functioning), and age, years of education and lesion size as predictor variables with accounting for all potential two-way and three-way interactions^19^. Given previous reports of higher MoCA scores in females^27^, the model was complemented by sex as additional predictor.

**Figure 3:**
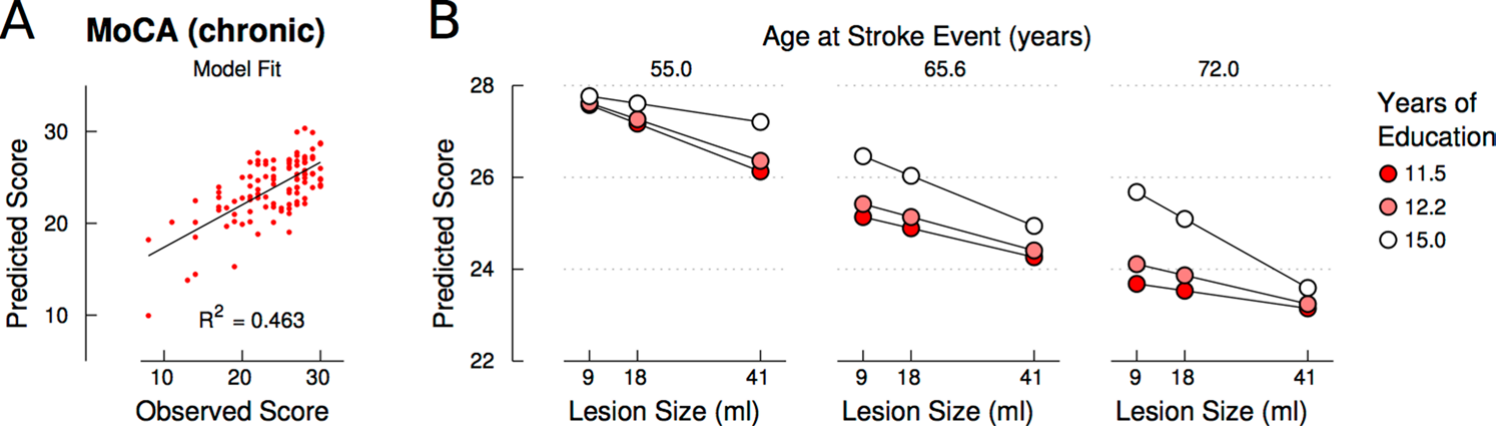
Years of education and age conjointly moderate the effect of lesion size on post-stroke cognitive outcome. (**A**) The model fit is illustrated by plotting observed against predicted scores. (**B**) Regression lines illustrate the significant three-way interaction of age, years of education, and lesion size in predicting chronic MoCA scores. Slopes are plotted at centerings for age, years of education, and lesion size at their respective 25th, 50th, and 75th percentiles. The figure was created using Matlab software, version 2018a https://www.mathworks.com/products/matlab.html and Inkscape, version 1.0 https://inkscape.org/.

Results revealed a significant fit of the overall model accounting for about 46% of variation in the chronic MoCA scores (F_(15,88)_ = 5.06, p < 0.001, R = 0.680, R^2^ = 0.463; Fig. 3A). By comparison, a reduced model without interaction effects accounted only for 36.7% of variance. In the full model, significant simple effects were observed for all three predictor variables, with age (p < 0.001) and lesion size (p < 0.003) being negatively and years of education (p = 0.034) positively associated with global cognitive functioning (see table e-2 for a detailed report). Whereas none of the two-way interactions approached significance (all p > 0.463), the significant three-way interaction (p = 0.049) indicated that effects of lesion size on post-stroke cognitive outcome were moderated by both age and years of education (Fig. 3B): In middle-aged stroke patients at ages around 66 years (Fig. 3B, middle panel), the well-established negative association between cognitive outcome and lesion size was found independent of educational attainment, which itself was positively associated with cognitive outcome. However, in young-aged stroke patients at ages around 55 years (Fig. 3B, left panel), the negative association between lesion size and cognitive functioning was substantially attenuated at higher years of education (see regression line with white markers), with higher-educated patients consistently achieving higher outcomes in the MoCA irrespective of lesion size, while middle- and lower-educated patients showed the known negative association between lesion size and cognitive outcome (see regression line with light red and dark red markers). In old-aged stroke patients at ages around 72 years (Fig. 3B, right panel), this pattern was reversed: Patients with low years of education generally showed low MoCA outcomes independent of lesion size (see regression line with dark red markers), whereas MoCA scores in patients with high educational attainment were negatively correlated with lesion size (see regression line with white markers). In other words, in old-aged stroke patients with low educational attainment even smaller lesions led to cognitive impairment. Finally, this three-way moderation effect was more pronounced in female than in male patients as indicated by the significant four-way interaction between age, years of education, lesion size, and sex (p = 0.022; figure e-1). However, although the matching procedure allowed controlling for confounds between the key variables of interest (i.e., lesion size, age, and education), female sex was correlated with larger lesion sizes (r = 0.229, p = 0.019; table e-3), which most likely has driven the four-way interaction.

Given the large variation in the duration of acute hospitalization and in the post-stroke time of the MoCA-assessment, we performed additional control analyses to test whether this may have biased the present analyses. However, including these variables in the statistical model as covariates did not change the results.

Taken together, larger lesion size was not invariably associated with poor cognitive outcome, but could rather be compensated for by concomitantly higher education and younger age. Conversely, even a small lesion size was associated with poor cognitive outcome in case of lower education and older age. The present data hence provide first evidence for a strong moderator effect not only of brain reserve, but also of an individual patient’s cognitive reserve on the often reported simple effect of lesion size.

### Prediction of stroke-induced impairment and disability

**Figure 4:**
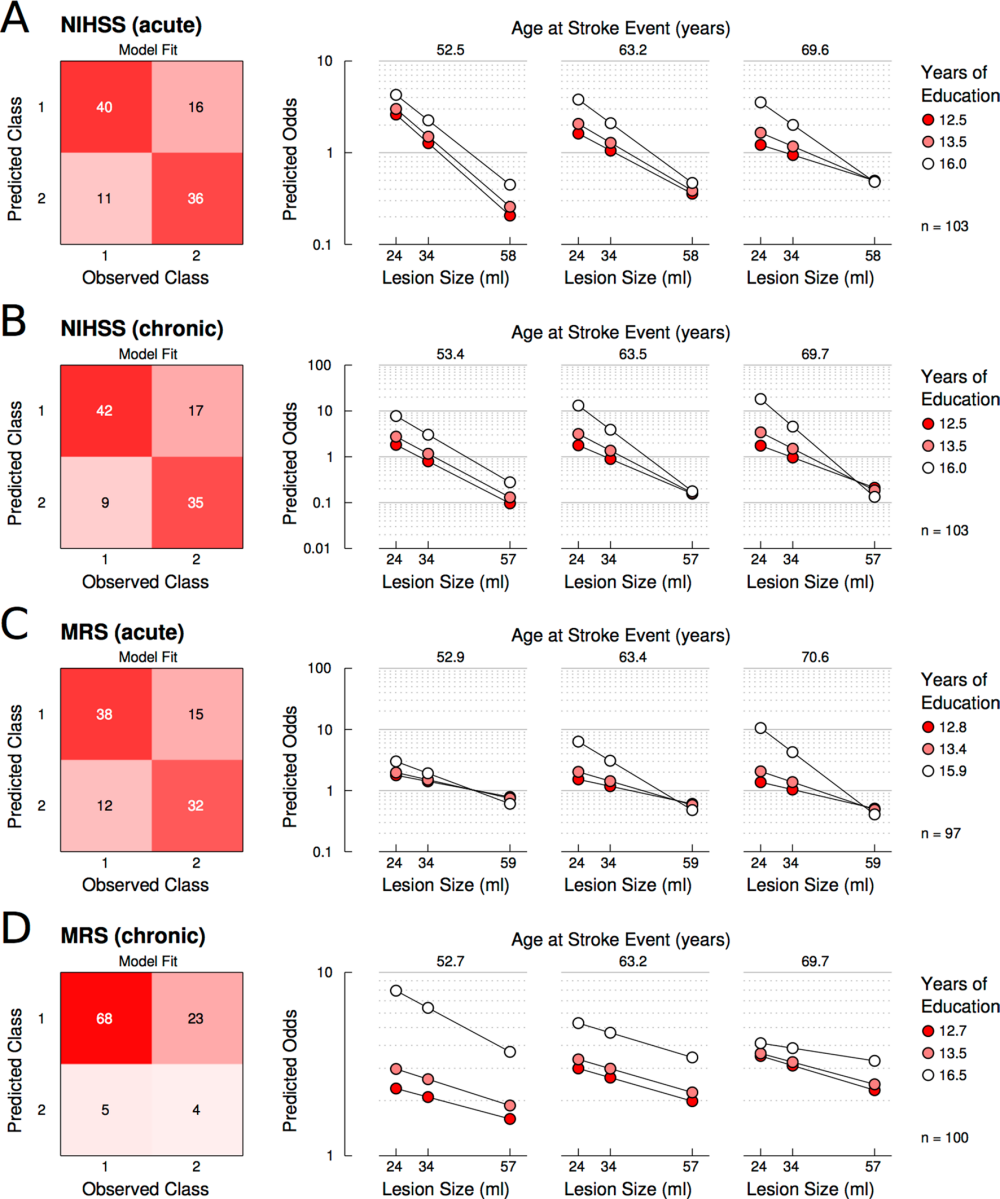
Years of education and age conjointly moderate the effect of lesion size on stroke-induced impairment (NIHSS) and disability (mRS). Three-way interactions of age, years of education, and lesion size were consistently observed for prediction of NIHSS in the acute (**A**) and the chronic stroke phase (**B**) as well as for prediction of mRS in the acute stroke phase (**C**) and a trend thereof in the chronic stroke phase (**D**). The left panels illustrate the model fit by plotting observed against predicted class assignments. The right panels illustrate the predicted odds of the probabilities for class 1 (favorable outcome) against class 2 (poor outcome) as a function of lesion size, age, and years of education. An odds of 1 would hence denote equal probabilities for better and poorer outcome, whereas an odds of 2 would reflect that the probability of a favorable outcome (class 1) is twice the size of the probability of a poor outcome (class 2). In turn, an odds of 0.5 would reflect that the probability of a favorable outcome (class 1) is half the size of the probability of a poor outcome (class 2). Slopes are plotted at centerings for age, education, and lesion size at their respective 25th, 50th, and 75th percentiles. Please note that predictor values corresponding to these percentiles vary slightly across panels due to slightly different sample sizes for clinical outcome scores (see “Results” section for details). The figure was created using Matlab software, version 2018a https://www.mathworks.com/products/matlab.html and Inkscape, version 1.0 https://inkscape.org/.

In the next step, we tested the generalizability of the hypothesis also in prediction of non-cognitive stroke deficits such as stroke-induced impairment measured by NIHSS and disability measured by mRS. We applied logistic regression analyses on dichotomized clinical outcome scores, which accounted for simple effects of age, years of education, and lesion size and for their potential two-way and three-way interactions. The model corroborated the key finding for the cognitive outcome: We consistently found significant three-way interactions for prediction of NIHSS in the acute (p = 0.035) and the chronic stroke phase (p = 0.010) as well as for prediction of mRS in the acute stroke phase (p = 0.019) and with a trend thereof in the chronic phase (p = 0.093, Fig. 4 and table e-2). With other words, the odds for better clinical outcome (class 1) did not solely depend on lesion size, but were further moderated by age and years of education. Particularly for NIHSS in older patients (Fig. 4A,B, right sub-panels), the odds for a better clinical outcome were higher for those with higher education and smaller lesions (see regression lines with white markers), whereas the odds for a better clinical outcome were generally lower for older patients with low educational attainment (see regression lines with dark red markers). No such beneficial effect of small lesion size and high education was evident in younger patients (Fig. 4A,B, left sub-panels). The same pattern was evident for the mRS in acute stroke (Fig. 4C). The odds for better outcome in the chronic mRS were highest for younger patients with higher educational attainment and smaller lesions (Fig. 4D). Thus, education and age conjointly moderated the negative impact of lesion size on stroke-induced impairment and disability both in the acute and chronic stroke phase.

## Discussion

The present study provides first direct evidence that (i) the concepts of brain and cognitive reserve (as approximated here by age and years of education) are applicable in acute pathology such as stroke and that (ii) their proxies mutually interact by beneficially moderating the detrimental effect of stroke-induced brain damage (i.e. lesion size) on both cognitive and clinical outcome.

### Interplay between years of education and age on effects of lesion size on post-stroke cognition

The key finding of the study is the interplay between years of education and age on effects of lesion size on post-stroke cognitive outcome as demonstrated by their significant three-way interaction. As of yet, their impact on post-stroke cognition has been only shown in isolation without taking their mutual interaction into account. Thus, the present findings go substantially beyond simple additive effects of education, age, and lesion size by revealing their non-additivity, i.e. interaction, which directly accounts for about 10% of inter-individual variability in cognitive post-stroke outcome. Thus, patients with a similar lesion load may present with a substantially different post-stroke cognitive outcome as the effects of stroke-induced brain damage depend systematically on a patient’s education and age (Fig. 3). The data uncover why and under which circumstances small lesions may lead to substantial cognitive decline^28^: In older patients with lower years of education, even small infarcts can entail a significant cognitive impairment, in contrast to patients with comparable age and lesion size but higher education. In turn, in younger patients, only large lesions lead to relevant cognitive impairment and only in the case of a lower years of education, whereas patients with higher education do not present with relevant cognitive deficits irrespective of lesion size. Due to the observed *interaction* effect, these results are not just an effect of age and age-related brain pathology in stroke outcome, as in this case a simple linear effect of age would be expected.

The mechanisms underlying the impact of years of education in stroke are likely to be similar to those established for cognitive reserve in neurodegeneration. Higher education is usually associated with better premorbid cognition, and it seems plausible that in higher-educated individuals stroke may lead to less severe cognitive impairment than in those with a lower educational attainment^29,30^. This might comprise a simple additive effect of cognitive reserve on post-stroke cognition. In line with the point, we demonstrated recently that years of education predicted both severity of education independent (alertness) and education dependent (working memory, executive functions, global cognition) cognitive deficits and disability (modified Rankin Scale) in the acute stroke phase^9^. However, as we have found the additional *non-additive* effect of cognitive reserve, it’s effect on stroke outcome appears to be more complex^31^ suggesting the existence of protective post-stroke mechanisms. Since higher cognitive reserve is linked to better networks compensation by pathology^6^, this may render subjects with higher cognitive reserve less vulnerable to damage per se. In addition, these patients may easier recruit undamaged network components that potentially increase rehabilitation efficacy. In other words, as most of the rehabilitation approaches are based on the learning of new behavioral strategies, a patient’s potential to respond to rehabilitation might at least to some extent depend on the individual cognitive reserve^8^ and, in turn, might therefore require more intensive attendance in patients with lower educational attainment. The moderator effect may however rely also on the complex interplay of several other factors, as for instance higher education (i.e. better cognitive reserve) is commonly associated with better socio-economic status, healthier lifestyle, and better management of vascular risk factors, therefore also promoting a better brain reserve^8^. Such an interdependency of cognitive and brain reserve has been demonstrated for several neural indices such as cortical thickness^32^, functional connectivity^33–35^, grey matter volume or its metabolism^36^. Thus, although we sought to minimize potential confoundings between education and age, indirect amplifications cannot be completely excluded. The four-way interaction with sex further indicated that the moderator effect of age and education on the negative relationship between lesion size and cognitive post-stroke outcome was more pronounced in female than in male patients. As this was most likely driven by a still present confound between female sex and larger lesion size, such equivocality has to be disentangled in future studies with larger samples that allow for more sophisticated matching strategies.

As previously discussed^8^, lesion load—its size and localization—also determines the compensational strategies and extent of neural compensation required: a small lesion of the association cortex might be well compensated by perilesional cortex^37^, but would require a shift in interhemispheric lateralization or recruitment of secondary functional centers if the primary functional center would suffered a stroke^37–39^. In many cases, more severe injuries in terms of size and strategical location require more compensatory events to maintain the function^40^. At the same time, such lesions compromise more neural reserve, as less brain tissue and fewer functional centers remain available, that constrains post-stroke neural compensation. Therefore, the neural compensation in stroke is a product of the interaction between premorbid brain and cognitive reserves and the severity of stroke-induced impairment^8^.

### Impact of years of education and age on stroke-induced impairment and disability

Years of education conjointly with age was found to moderate also the impact of lesion size on stroke impairment and disability both in the acute and chronic phase of stroke. To the best of our knowledge, this is the first time that such kind of interaction and, in particular, a role of years of education on clinical outcome measures has been demonstrated. The effect of educational attainment on stroke outcome has been barely investigated until now. Educational history was independently associated with favorable post-stroke survival independent of age^41^, though the study did not analyze lesion load. The underlying mechanisms are most likely the same as discussed above for the cognitive post-stroke outcome. It is to highlight, however, that the presence of non-additive effect of cognitive reserve on NIHSS and mRS scores suggests its post-stroke protective impact on stroke impairment and disability both in the acute and the chronic stroke phase. The present findings hence demonstrate that prediction of stroke outcome benefits from considering not only lesion characteristics^42^. The specific lesion anatomy is particularly essential for domains relying on circumscribed brain networks such as motor and language^43,44^. Stroke outcome represents however not just a sum of the distinct functional deficits but rather a global measure of compensation and adaptation to the lesion—features that are only partly covered by lesion factors and age. Though the other proxies of cognitive reserve (e.g. occupation, life style determinants) are still to be elucidated for stroke, we argue that the concepts of cognitive and brain reserve represent a valuable theoretical framework to capture the increasing inter-individual variability across patients and to implement many factors that might impact stroke outcome. Inclusion of additional factors such as severity of leukoaraiosis or brain atrophy may further improve the model’s prediction value but would also require larger sample sizes.

### Caveats and limitations

As for any other patient study, the clinical cohort influences the findings: The study procedure may have introduced a selection bias towards patients with less severe stroke and more favorable stroke outcome as patients had to be in a condition to attend the assessments in the chronic stage. Future studies are required to explore the here reported interaction effects in patients with severe stroke, e.g. after a (sub)total infarct in the middle cerebral artery territory. Furthermore, patient groups with low versus high cognitive reserve were not explicitly matched but only controlled for lesion hemisphere, given that MoCA-scores may be lower for the left hemisphere stroke^30,45^. Future studies may therefore explore the influence of the lateralization and also the anatomy of the lesion on the here established effects. Another limitation concerns years of education, which do not perfectly represent cognitive reserve, as it does not include other important aspects such as lifetime intellectual activities, lifestyle or occupational history, considering that individuals with limited education may build cognitive reserve in other ways across the lifespan. Education might also be linked to health outcomes via multiple pathways (e.g. socio-economic status and access to health care)^46^, and not just those claimed to represent cognitive reserve mechanisms. However, information on years of education is much easier to acquire in routine clinical practice that makes data obtainable especially in the acute setting. In Germany, where the study has been performed, the patient care and stroke treatment are standardized and independent from socio-economic status. In addition, the research on cognitive reserve in neurodegeneration was established using years of education as a main proxy^47^. There is also sufficient evidence for its legitimate usage as a valid, albeit rough measure of cognitive reserve^48^, though future research of other potential proxies of cognitive reserve in stroke is required. Finally, we used age as proxy of brain reserve in contrast to more common operationalization in neurodegeneration studies as, for instance, maximum attained brain size or brain size. However, as argued above, age is associated with several neuroanatomical determinants for a patient’s susceptibility to stroke-induced impairment^10–12^, easy to acquire in clinical settings and is a commonly used proxy in stroke research to capture the inter-individual variability. In addition, the term “reserve” applies to additionally available brain resources, whereas age is rather inversely associated with brain volumetric characteristics.

## Outlook and conclusion

Present findings provide several implications for future stroke research. Current studies on stroke recovery are seeking advanced neuroimaging biomarkers^37,49,50^. The present results demonstrate that if confounds between simple proxies of brain and cognitive reserve such as age and years of education are controlled for and their interaction with stroke-induced brain damage is explicitly modeled, a considerable amount of inter-individual variance in cognitive and functional post-stroke outcome can already be predicted. The applicability of the concepts of brain and cognitive reserve in stroke suggests a new theoretical framework for prediction of stroke outcome that may easily accommodate further additional proxies. Altogether, the non-additive effect of cognitive and brain reserve implies their conjoint post-stroke protective impact on stroke-induced cognitive deficits, impairment, and disability. Finally, better understanding of patients’ variability in stroke outcome improves its prediction and might further help to establish the individualized rehabilitation approaches.

## Supplementary Information

Supplementary Information

## Data Availability

Individual patient data will not be distributed openly to conform to the data privacy statement signed by patients included in the study. Specific aspects of the anonymized raw data will be shared upon request.
