# Supplementary material for: Interaction between cognitive reserve and age moderates effect of lesion load on stroke outcome
Source: Sci Rep. 2021 Feb 24;11:4478. doi: 10.1038/s41598-021-83927-1 (PMC7904829; doi:10.1038/s41598-021-83927-1)
Supplement: Supplementary file 1 — Supplementary Information [file 41598_2021_83927_MOESM1_ESM.docx]

**Interaction between cognitive reserve and age moderates effect of lesion load on stroke outcome**

**Cover title:** **Protective effect of years of education**

Roza M. Umarova MD*^1,2,3,4^, Lena V. Schumacher PhD^1,5^, Charlotte S.M. Schmidt PhD^1,3^,
Markus Martin MD^1,3,6^, Karl Egger MD^1,7^, Horst Urbach MD^1,7^, Jürgen Hennig PhD^1,3,8^, Stefan Klöppel MD^1,2,3,9^, Christoph P. Kaller PhD*^1,3,7^

^1^ Faculty of Medicine, University of Freiburg, Germany | ^2^ Department of Psychiatry and Psychotherapy, Medical Center – University of Freiburg, Germany | ^3^ BrainLinks-BrainTools Cluster of Excellence, University of Freiburg, Germany | ^4^ Department of Neurology, Inselspital, University of Bern, Switzerland | ^5^ Medical Psychology and Medical Sociology, Faculty of Medicine, University of Freiburg, Germany | ^6^ Center for Geriatric Medicine and Gerontology, Medical Center – University of Freiburg, Germany | ^7^ Department of Neuroradiology, Medical Center – University of Freiburg, Germany | ^8^ Medical Physics, Department of Radiology, Medical Center – University of Freiburg, Germany | ^9^ University Hospital of Old Age Psychiatry Bern, Switzerland

**SUPPLEMENTAL MATERIAL**

**Table e-1.** Sample characteristics in stroke patients with low and high educational attainments.

|  | Lower years of education (n=52) | |  | Higher years of education (n=52) | |  | Group Comparison | | | |
| --- | --- | --- | --- | --- | --- | --- | --- | --- | --- | --- |
| Variable | Mean ± SD | Range |  | Mean ± SD | Range |  | Stat(df) | Value | p |  |
| Years of Education | 11.2 ± 1.0 | 8.0-12.0 |  | 15.8 ± 2.9 | 13-23 |  | t(102) | 10.564 | <0.001 |  |
| Age (years) | 63.9 ± 12.5 | 28.8-84.6 |  | 62.7 ± 11.9 | 31.3-85.6 |  | t(102) | 0.496 | 0.621 |  |
| Sex (f/m) | 16 / 36 |  |  | 16 / 36 |  |  | χ^2^(1) | 0 | >0.999 |  |
| Lesion Size (ml) | 33.6 ± 37.0 | 1.0-33.3 |  | 33.6 ± 35.8 | 0.3-141.2 |  | t(102) | -0.005 | 0.996 |  |
| NIHSS at admission | 7.7 ± 5.6 | 0-18 |  | 7.7 ± 6.1 | 0-22 |  | t(100) | 0.141 | 0.888 |  |
| Stroke Lateralization (Left/Right) | 35 / 17 |  |  | 28 / 24 |  |  | χ^2^(1) | 1.973 | 0.160 |  |
| Thrombolysis (yes/no) | 26 / 26 |  |  | 32 / 20 |  |  | χ^2^(1) | 1.403 | 0.236 |  |
| Duration of acute hospitalization (days) | 10.0 ± 4.0 | 4-21 |  | 9.2 ± 3.6 | 4-18 |  | t(102) | 1.086 | 0.280 |  |
| Time of chronic assessment (months post stroke) | 16.7 ± 18.3 | 5.2-65.9 |  | 17.4 ± 18.2 | 5.0-72.5 |  | t(102) | 0.183 | 0.855 |  |
| Total Brain Volume (ml) | 1171± 127 | 964-1529 |  | 1159±111 | 945-1444 |  | t(102) | 0.506 | 0.609 |  |

N.B. SD, standard deviation; Stat, statistic; df, degrees of freedom; f/m, female/male; l/r, left/right; ml, milliliter.

**Table e-2.** Overview of estimated model parameters.

| Criterion | Predictor | b | SE | t | p |
| --- | --- | --- | --- | --- | --- |
| MoCA (chronic) | Intercept | 24.3212 | 0.4652 | 52.2793 | 0.0000 |
|  | L (lesion size) | -0.0389 | 0.0129 | -3.0166 | 0.0033 |
|  | A (age) | -0.1885 | 0.0454 | -4.1545 | 0.0001 |
|  | E (education) | 0.3660 | 0.1704 | 2.1484 | 0.0344 |
|  | S (sex) | 2.1244 | 0.9304 | 2.2833 | 0.0248 |
|  | L:A | -0.0006 | 0.0010 | -0.6194 | 0.5372 |
|  | L:E | -0.0008 | 0.0044 | -0.1922 | 0.8481 |
|  | A:E | 0.0064 | 0.0165 | 0.3886 | 0.6985 |
|  | A:S | -0.0035 | 0.0908 | -0.0389 | 0.9691 |
|  | L:S | 0.0063 | 0.0258 | 0.2460 | 0.8062 |
|  | E:S | -0.2514 | 0.3407 | -0.7378 | 0.4626 |
|  | L:A:E | -0.0006 | 0.0003 | -1.9905 | 0.0496 |
|  | L:A:S | -0.0004 | 0.0021 | -0.1902 | 0.8496 |
|  | A:E:S | -0.0146 | 0.0329 | -0.4436 | 0.6584 |
|  | L:E:S | -0.0037 | 0.0088 | -0.4155 | 0.6788 |
|  | L:A:E:S | -0.0014 | 0.0006 | -2.3406 | 0.0215 |
| NIHSS (acute) | Intercept | -0.4757 | 0.3320 | -1.4329 | 0.1519 |
|  | L (lesion size) | -0.0591 | 0.0161 | -3.6613 | 0.0003 |
|  | A (age) | 0.0127 | 0.0279 | 0.4562 | 0.6482 |
|  | E (education) | 0.1348 | 0.0928 | 1.4534 | 0.1461 |
|  | L:A | 0.0015 | 0.0012 | 1.2416 | 0.2144 |
|  | L:E | -0.0034 | 0.0043 | -0.8024 | 0.4223 |
|  | A:E | -0.0081 | 0.0096 | -0.8443 | 0.3985 |
|  | L:A:E | -0.0007 | 0.0003 | -2.1051 | 0.0353 |
| NIHSS (chronic) | Intercept | -0.9476 | 0.4402 | -2.1526 | 0.0313 |
|  | L (lesion size) | -0.1033 | 0.0276 | -3.7478 | 0.0002 |
|  | A (age) | 0.0032 | 0.0327 | 0.0978 | 0.9221 |
|  | E (education) | 0.2007 | 0.1143 | 1.7564 | 0.0790 |
|  | L:A | -0.0010 | 0.0016 | -0.6486 | 0.5166 |
|  | L:E | -0.0133 | 0.0072 | -1.8603 | 0.0628 |
|  | A:E | -0.0163 | 0.0117 | -1.3983 | 0.1620 |
|  | L:A:E | -0.0013 | 0.0005 | -2.5655 | 0.0103 |
| mRS (acute) | Intercept | -0.1505 | 0.2969 | -0.5070 | 0.6121 |
|  | L (lesion size) | -0.0468 | 0.0145 | -3.2206 | 0.0013 |
|  | A (age) | -0.0107 | 0.0218 | -0.4909 | 0.6235 |
|  | E (education) | 0.0430 | 0.1157 | 0.3717 | 0.7101 |
|  | L:A | -0.0015 | 0.0009 | -1.6715 | 0.0946 |
|  | L:E | -0.0136 | 0.0055 | -2.4686 | 0.0136 |
|  | A:E | 0.0075 | 0.0097 | 0.7724 | 0.4399 |
|  | L:A:E | -0.0008 | 0.0003 | -2.3303 | 0.0198 |
| mRS (chronic) | Intercept | 1.1100 | 0.2517 | 4.4096 | 0.0000 |
|  | L (lesion size) | -0.0141 | 0.0067 | -2.0975 | 0.0360 |
|  | A (age) | 0.0005 | 0.0209 | 0.0238 | 0.9810 |
|  | E (education) | 0.1696 | 0.0988 | 1.7168 | 0.0860 |
|  | L:A | 0.0005 | 0.0005 | 1.0896 | 0.2759 |
|  | L:E | -0.0008 | 0.0023 | -0.3433 | 0.7314 |
|  | A:E | -0.0097 | 0.0092 | -1.0573 | 0.2904 |
|  | L:A:E | 0.0003 | 0.0002 | 1.6778 | 0.0934 |

N.B. b, unstandardized regression coefficient; SE, standard error. MoCA, Montreal Cognitive Assessment; NIHSS, National Institutes of Health Stroke Scale; mRS, modified Rankin Scale. A, age (years); L, lesion volume (ml); E, education (years); S, sex (female = 1, male = 0). Parameters are reported for lesion size, age, and education centered at their respective means. Sex is centered at 0.5 thus reflecting sex-independent parameter estimates. Colons denote product terms (interaction effects). Significant effects (p < .05) or trends thereof (p < .10) are highlighted in light gray.

**Table e-3.** Bivariate correlations between predictors lesion size, age, education, and sex.

|  | L | A | E | S |
| --- | --- | --- | --- | --- |
| L (Lesion size |  | -.141 | .022 | **.229*** |
| A (Age) | .155 |  | -.031 | -.056 |
| E (Education | .824 | .754 |  | -.144 |
| S (Sex) | **.019** | .572 | .145 |  |

N.B. Pearson correlations (r) are reported in the cells above the diagonal whereas the respective p-values are reported in the corresponding cells below the diagonal.


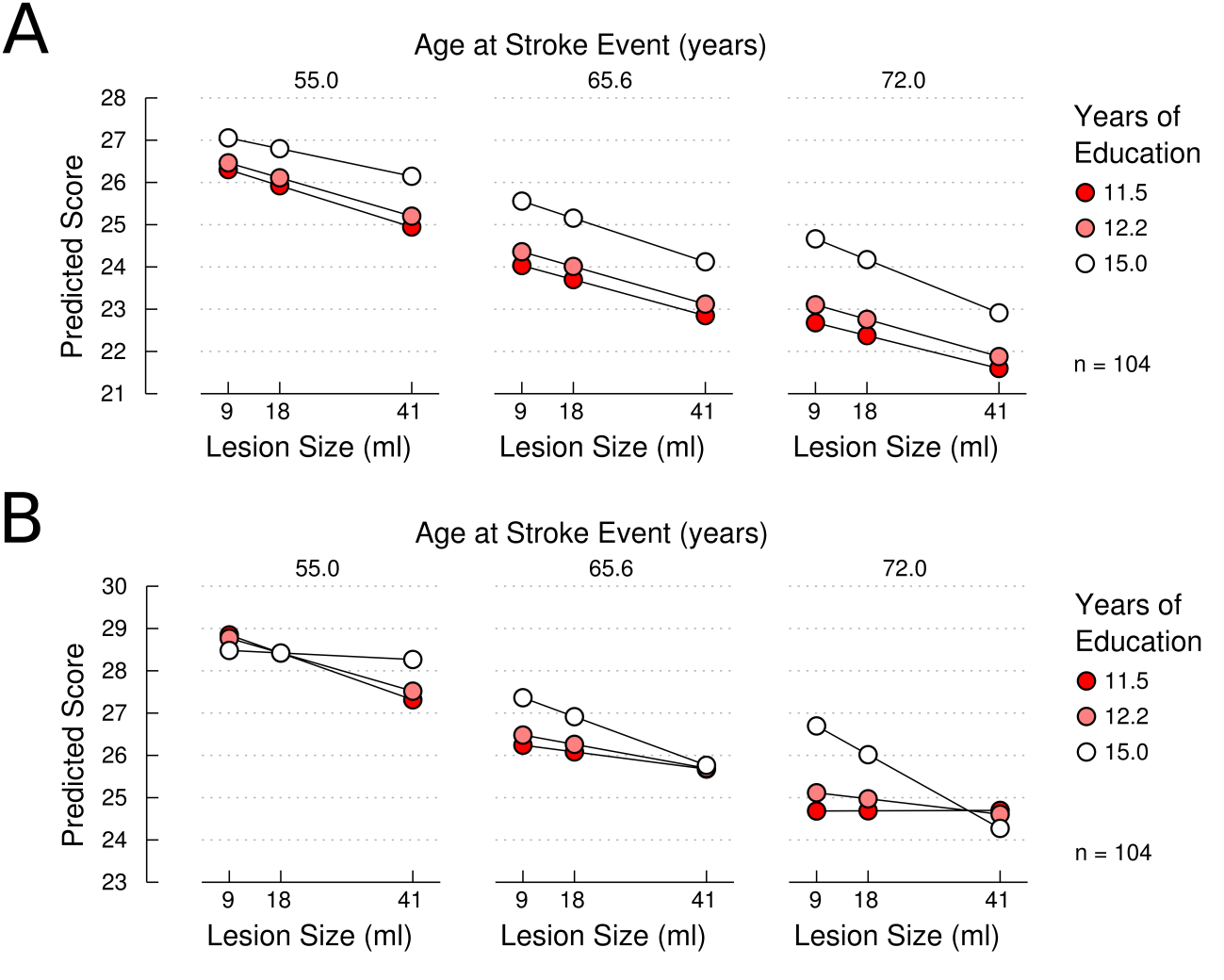


**Figure e-1.** The four-way interaction of lesion size, age, education, and sex. The three-way interaction of age, education, and lesion size in predicting chronic MoCA scores was more pronounced in (B) female than in (A) male patients. Slopes are plotted at centerings for age, education, and lesion size at their respective 25^th^, 50^th^, and 75^th^ percentiles. Sex is differentially centered in panels (A) and (B) at values for male and female sex, respectively. The figure was created using Matlab software, version 2018a https://www.mathworks.com/products/matlab.html and Inkscape, version 1.0 https://inkscape.org/.
